# Effects of Statins on Cardiorenal Syndrome

**DOI:** 10.1155/2012/162545

**Published:** 2012-06-26

**Authors:** Shusuke Yagi, Ken-ichi Aihara, Yasumasa Ikeda, Masashi Akaike, Masataka Sata, Toshio Matsumoto

**Affiliations:** ^1^Department of Cardiovascular Medicine, The University of Tokushima Graduate School of Health Biosciences, 3-18-15 Kuramoto-cho, Tokushima 770-8503, Japan; ^2^Department of Medicine and Bioregulatory Sciences, The University of Tokushima Graduate School of Health Biosciences, 3-18-15 Kuramoto-cho, Tokushima 770-8503, Japan; ^3^Department of Pharmacology, The University of Tokushima Graduate School of Health Biosciences, 3-18-15 Kuramoto-cho, Tokushima 770-8503, Japan; ^4^Department of Medical Education, The University of Tokushima Graduate School of Health Biosciences, 3-18-15 Kuramoto-cho, Tokushima 770-8503, Japan

## Abstract

Cardiovascular disease and renal disease have a close relationship that forms a vicious cycle as a cardiorenal syndrome (CRS). Oxidative stress, endothelial dysfunction, and vascular inflammation could be therapeutic targets when the renin-angiotensin-aldosterone system is activated by accumulation of conventional cardiovascular risk factors; however, a strategy for management of CRS has not been established yet. Statins, HMG-CoA reductase inhibitors, have not only cholesterol-lowering effects but also pleiotropic effects on cardiovascular systems, including anti-inflammatory and antioxidant effects and improvement of nitric oxide bioavailability. Since recent studies have indicated that statins have beneficial effects on chronic kidney disease and heart failure as well as coronary artery disease in cholesterol-lowering-dependent/independent manners, treatment with statins might be a successful strategy for preventing deterioration of CRS.

## 1. Introduction

It is well known that metabolic syndrome contributes directly to the occurrence of cardiovascular events. Accumulation of cardiovascular risk factors, including dyslipidemia, hypertension, and diabetes, activates the renin-angiotensin-aldosterone system (RAAS), leading to not only ischemic cardiovascular disease (CVD) but also left ventricular (LV) dysfunction and chronic kidney disease (CKD) [1]. It is also known that LV dysfunction and renal dysfunction frequently coexist in the same individual. Therefore, it has been advocated that cardiovascular disease and renal disease are closely related to each other as a cardiorenal syndrome (CRS). Disorders of these two organs are co activated and co regulated by various lifestyle-related problems, falling into a vicious cycle of cardiorenal diseases; however, a strategy for management of this syndrome has not been established yet. Therefore, identification of the key therapeutic targets of CRS is needed to break the vicious cycle development of cardiac disease and renal disease. 

Statins, HMG-CoA reductase inhibitors, have not only cholesterol-lowering effects but also pleiotropic effects on cardiovascular systems, including anti-inflammatory and antioxidant effects and improvement of nitric oxide (NO) bioavailability [2–5]. In this paper, we focus on the effects of statins on CRS. 

## 2. CRS Is Based on a Close Network between Heart and Kidney Disorders

CRS is defined as “disorders of the heart and kidneys whereby acute or chronic dysfunction in one organ may induce acute or chronic dysfunction of the other” [6]. Since low cardiac output due to heart failure (HF) leads to decrease in renal blood flow, HF exacerbates renal function. In fact, a 25% decrease in cardiac output leads to a 50% decrease in renal blood flow, and 25% to 40% of patients with decompensated HF experience deterioration in renal function [7, 8]. Renal perfusion is strictly regulated by an autoregulation system to maintain intraglomerular pressure at a constant level; however, prolonged HF evokes glomerular endothelial dysfunction and breakdown of the glomerular autoregulation system. Decrease in renal perfusion due to HF activates the RAAS, leading to cardiorenal damage. It has been reported that patients with CKD, especially elderly patients, are at high risk for major CVD morbidity and mortality. Indeed, the mortality rate in a 2-year interval after acute myocardial infarction is around 50% in patients with stage 5 CKD. In addition, it is noteworthy that patients with CKD have a 10- to 20-fold increased risk of cardiac death [9, 10]. Albuminuria, an early manifestation of CKD, has been shown to be a risk factor for not only the development of CKD but also cardiovascular disease even at stage 1 of CKD [11]. Therefore, CKD is characterized as intraglomerular hypertension and glomerular leakage from albuminuria to overt proteinuria, leading to decrease in glomerular filtration rate (GFR) and ultimately end-stage kidney disease. 

Taken together, the heart and kidneys form a tight network in both physiological and pathological conditions, and homeostatic failure of these organs exacerbates clinical states of CKD and CVD in a synergistic manner. 

## 3. Mechanisms of the Development of CRS

**Figure 1:**
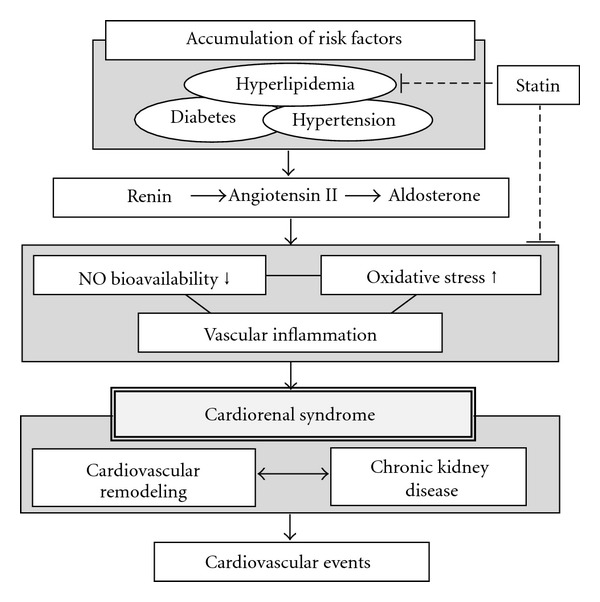
Activation of the renin-angiotensin-aldosterone system due to accumulation of cardiovascular risk factors causes imbalance between nitric oxide (NO) bioavailability and oxidative stress, leading to vascular inflammation. This upstream signaling exacerbates cardiorenal syndrome and evokes cardiovascular events. Statins ameliorate not only the serum lipid profile but also oxidative stress, NO bioavailability, and vascular inflammation by their pleiotropic effects.

The mechanisms of the development of CVD evoked by CKD have not been fully clarified; however, a number of previous studies suggested that several biomarkers yield clues for understanding the mechanisms of the development of CRS. Under normal physiological conditions, oxidative stress and NO bioavailability are each countervailed to maintain vascular homeostasis; however, under pathological conditions such as excessive RAAS activation, this balance is disrupted by an increase of oxidative stress and decrease of NO bioavailability, and disruption of the balance evokes vascular inflammation, leading to cardiovascular remodeling and CKD and eventually to cardiovascular events (Figure 1). Therefore, oxidative stress and NO bioavailability and vascular inflammation could be therapeutic targets for preventing cardiovascular events in addition to conventional risk factors of CVD such as hypertension, diabetes, dyslipidemia, and smoking. 

## 4. Statins Improve Endothelial Function

Endothelial function is important for maintaining vascular homeostasis. Endothelium-derived NO mediates vascular relaxation and inhibits platelet aggregation, vascular smooth muscle proliferation, and endothelium-leukocyte interactions and it protects the cardiovascular system from remodeling [12–14]. On the other hand, accumulation of cardiovascular risk factors impairs endothelial function, which is the earliest manifestation of atherosclerosis. It has been recognized that improvement of endothelial function by statins was observed before a significant reduction in serum cholesterol level, suggesting that these preferable effects of statins on endothelial function are cholesterol-lowering-independent actions [15, 16]. It has been shown that statins increase NO bioavailability by activation and up regulation of endothelial NO synthase (eNOS) through inhibition of Rho/ROCK signaling, activation of the PI3kinase/Akt pathway, and amelioration of eNOS uncoupling [4, 17–21]. 

## 5. Statins Decrease Oxidative Stress

Another potential mechanism by which statins may improve endothelial function is their antioxidant effects. Reactive oxygen species (ROS) as a major source of oxidative stress are mainly produced by NADPH oxidase in the cardiovascular system, and Rac-1, a small G protein, has recently been shown to be a key molecule for the assembly and function of NADPH oxidase components [22]. Therefore, NADPH oxidase components, especially Rac-1, could be targets of statin therapy to reduce oxidative stress. In fact, statins attenuate angiotensin II-induced oxidative stress in cardiac myocytes and vascular smooth muscle cells by inhibiting Rac-1-mediated NADH oxidase activity [22, 23]. Statins also reduce mRNA expression of p22^phox^ and Nox1, NADPH oxidase subunits [3]. 

## 6. Statins Ameliorate Vascular Inflammation

It is well known that vascular inflammation plays a central role in the pathogenesis of cardiovascular disease. In addition, it is a widely accepted view that atherosclerosis is a chronic inflammatory disease that is initiated by endothelial cell dysfunction at the vascular surface [24]. Inflammatory cytokines secreted from mononuclear cells such as macrophages and T lymphocytes promote endothelial dysfunction, smooth muscle cell proliferation, and thrombosis. An early step in atherogenesis involves monocyte adhesion to the endothelium and penetration into the subendothelial space. It has been shown that statins reduce expression levels of proinflammatory cytokines and adhesion molecules, including tumor necrosis factor- (TNF)-*α*, interleukin (IL)-1*β* and IL-6, soluble vascular cell adhesion molecule-1 (sVCAM-1), and von Willebrand factor, and that statins reduce the number of inflammatory cells in atherosclerotic plaques [2, 25–27]. More recently, it has been shown that statins inhibit endothelial exocytosis of Weibel-Palade bodies, endothelial cell granules containing adhesion molecules, in an eNOS-dependent manner and that statins attenuate T cell-mediated myocardial inflammation in a KLF2-dependent manner in a mouse model [27, 28]. The inhibitory effects on endothelial exocytosis and modulation of T-cell function in the immune system may explain the mechanisms of the anti-inflammatory effects of statins in addition to improvement of imbalance between NO bioavailability, and oxidative stress.

Taken together, the results indicate that administration of statins could be a sound strategy for CRS patients with excessive oxidative stress, decreased NO bioavailability and vascular inflammation.

## 7. Statins for CVD

As for primary prevention against CVD, initial trials of lipid-lowering with clofibrate, cholestyramine, and gemfibrozil failed to reduce coronary mortality [29–31]; however, WOSCOPS [32], a landmark trial, showed that pravastatin reduced coronary mortality and the following AFCAPS/TexCAPS trial revealed that lovastatin reduced cardiovascular events [33]. In addition, the MEGA trial showed that Japanese hypercholesterolemia patients with no history of coronary disease or stroke, relatively low-risk group for CVD, could benefit from pravastatin for primary prevention against CVD [34]. On the other hand, the 4S and LIPID trials demonstrated that statin therapy improved total mortality as well as morbidity as secondary prevention of CVD [35–37]. 

The ASTEROID and REVERSAL trials showed that intensive statin therapy in patients with CVD reduced coronary atherosclerotic volume dependent on levels of LDL-C reduction [38, 39]. The PROVE-IT and TNT trials indicated that intensive statin therapy prominently decreased cardiovascular events compared with standard statin therapy in patients with acute or stable CVD, respectively [40, 41]. 

## 8. Statins for Systolic HF

The end-stage manifestation of cardiac remodeling due to ischemic and non ischemic heart diseases leads to systolic HF, and it had been expected that statins would be effective for preventing systolic HF. Experimental studies and a meta-analysis showed that a statin ameliorates cardiac remodeling and systolic function [42, 43]; however, two major randomized trials, CORONA and GISSI-HF, failed to reveal beneficial effects of rosuvastatin in patients with systolic HF [44, 45]. These two studies included patients of relatively advanced age who had severe systolic heart failure with/without ischemic heart disease. The CORONA study included patients with ischemic systolic heart failure (mean age of 73 y.o, NYHA class II to IV, mean LV ejection fraction of 31%), and the GISSI-HF study included over 60% of subjects with non-ischemic heart disease (mean age of 68 y.o. NYHA class II to IV, mean LV ejection fraction of 33%), suggesting that a statin is not effective for severe systolic HF in patients with both ischemic and non-ischemic heart diseases. Post-hoc analysis of the results of these studies showed that a statin was effective with patients in a low N terminal-pro B type natriuretic peptide (NT-proBNP) group (<103 pmol/L: 868 pg/mL) and in a high-sensitivity CRP (hs-CRP) group (> or = 2.0 mg/L) [46, 47]. These results indicate that statin treatment is effective in patients with early-stage cardiac injury but not in patients with advanced-stage cardiac injury and that statin therapy is more beneficial in HF patients with increased hs-CRP. 

## 9. Statins for Diastolic HF

LV diastolic dysfunction is an early manifestation of cardiac remodeling, and statins prevent cardiac remodeling including cardiac fibrosis and hypertrophy. Therefore, there is a possibility that statins improve LV diastolic function. Several experimental and observational studies showed the effectiveness of a statin for LV diastolic function [48–51]. In addition, a randomized controlled small trial of statin therapy in patients with diastolic HF (statin group, *n* = 81; no statin group, *n* = 189) showed that a statin improved survival in patients with diastolic HF during a 5-year follow-up period [52]. Since other antioxidant agents such as RAAS inhibitors have some beneficial effects on cardiac diastolic function, there is a possibility that statins might work for improving cardiac diastolic function via attenuation of oxidative stress. 

## 10. Statins for Atrial Function

Elevated cardiac end-diastolic pressure due to cardiac diastolic dysfunction plays a central role in atrial remodeling, leading to atrial fibrillation (AF) and cardiogenic embolism. Although antiarrhythmic and anticoagulant agents are used for these pathological conditions, these drugs are not able to ameliorate atrial remodeling. It has been reported that structural and electrophysiological changes in the atrium are associated with inflammation and oxidative stress [53]. It has also been reported that statins reduce the incidence of AF through attenuation of atrial remodeling [54]. A meta-analysis suggested an antiarrhythmic effect of statins against AF, especially under conditions of increased inflammation such as post-operative cardiac surgery and acute coronary syndromes [54]. These studies indicate that statin therapy prevents not only LV remodeling but also atrial structural and electrical remodeling, leading to decrease in the incidence of AF and cardiogenic embolism. 

## 11. Statins for CVD in Patients with CKD

Mild to moderate CKD is associated with increased cardiovascular risk. Therefore, strict management of serum lipid profile by statins also seems to be effective for reducing cardiovascular events in patients with mild to moderate CKD. A meta-analysis showed that statins reduce all-cause mortality, cardiovascular death, and non fatal cardiovascular events in mild to moderate CKD patients [55]. Post-hoc subgroup analysis of the Treating to New Targets (TNT) trial showed that a high dose of atorvastatin (80 mg) reduced cardiovascular events more than did a low dose of atorvastatin (10 mg) [56].

Although the SHARP trial, a randomized large-scale trial, has directly shown that simvastatin plus ezetimibe reduced the incidence of major vascular events in patients with CKD compared with placebo, the 4-D, AURORA, and SHARP trials showed no definite clinical benefit of statin monotherapy in hemodialysis patients regardless of significant reductions in LDL-C [57, 58]. These findings indicate that a statin is no longer able to reduce cardiovascular events in end-stage CKD, and statin therapy should therefore be recommended to CKD patients with mild to moderate CKD.

## 12. Statins for Renal Function

Experimental studies showed that hyperlipidemia is a risk factor for renal disease: cholesterol loading enhances glomerular injury and the decrease in serum lipid level by statins slows the rate of renal injury progression [59, 60]. Deposition of lipids in glomeruli and activation of the RAAS by accumulation of CVD risk factors cause glomerular endothelial dysfunction and increase intraglomerular pressure, leading to tangible albuminuria, an early manifestation of proteinuria, and decrease in glomerular filtration rate (GFR). Since albuminuria also directly injures glomeruli by itself, reduction of albuminuria might be essential to prevent progression of CKD. Animal studies have shown renal protective actions of statins [61, 62]; however, there are conflicting data concerning the effects of statins on renal function. In a clinical setting, two meta-analyses have shown that statins decrease albuminuria [63, 64]; whereas, other studies have shown that there were no significant effects of statins on albuminuria [65–67]. Additional evidence is needed to apply statin administration for treatment of CKD.

## 13. Beneficial Effects of Pitavastatin, a New ****Lipophilic Strong Statin

It has not been established whether the beneficial effects of statins on cardiorenal tissues are class effects; however, lipophilic statins seem to have direct effects on cardiovascular organs. The newly developed drug pitavastatin, a lipophilic and a strong statin, is effective for lowering LDL-cholesterol and increasing high-density lipoprotein cholesterol (HDL-C), which are preferable in the serum lipid profile for preventing CVD. In terms of tissue distribution of statins, pharmacokinetic profile before and after liver metabolism is an important consideration. Most of pitavastatin is absorbed following ingestion (80%), and its level of protein binding is extremely high (>95%) [68]. In addition, pitavastatin is hardly metabolized by cytochrome p450 (CYP), and most of the bioavailable pitavastatin is excreted in an unchanged form in bile and enters enterohepatic circulation by reabsorption in the small intestine [69]. Therefore, bioavailable pitavastatin is able to be distributed directly to cardiovascular and renal tissues directly and exerts cardiorenal protective effects. From these facts, pitavastatin has been expected to have stronger pleiotropic effects than those of other classical statins. In fact, it has been reported that pitavastatin has anti-oxidant and anti-inflammatory effects and that it improves NO bioavailability, leading to improvement of cardiorenal function [70–78]. Minimal interaction with CYP suggests that pitavastatin is safer than other statins regardless of lipophilicity.

## 14. Pitavastatin Ameliorates CRS 

Our previous experimental data showed that pitavastatin treatment improved angiotensin II-induced LV remodeling, renal insufficiency, atrial remodeling, incidence of AF, and atrial prothrombotic condition in eNOS knockout mice [79, 80]. In that study, we concluded that pitavastatin exerts eNOS-independent protective actions against angiotensin II-induced cardiovascular remodeling and renal insufficiency through inhibition of the transforming growth factor- (TGF)-*β* 1-Smad 2/3 signaling pathway by suppression of oxidative stress [79, 80]. In addition, our clinical study showed that a low dose (1 mg/day) of pitavastatin improved LV diastolic function and reduced albuminuria [81]. In that study, statistical analysis demonstrated that the effect of pitavastatin on cardiorenal protection was associated with reduction of oxidative stress but not reduction of LDL-C [81].

 Pitavastatin might have more overt pleiotropic effects, especially on reduction of oxidative stress, than those of other statins. A large clinical trial is needed to confirm the effects of pitavastatin on CRS.

## 15. Conclusion

Accumulation of CVD risk factors leads to activation of the RAAS, which exacerbates CRS with inflammation and eventually evokes cardiovascular events. Statin therapy plays an important role in prevention of these cardiovascular and renal events. Early use of statins is recommended in patients with multiple cardiovascular risk factors before the development of irreversible cardiorenal remodeling.
